# A Bayesian Approach for Decision Making on the Identification of Genes with Different Expression Levels: An Application to *Escherichia coli* Bacterium Data

**DOI:** 10.1155/2012/953086

**Published:** 2012-03-05

**Authors:** Erlandson F. Saraiva, Francisco Louzada, Luís A. Milan, Silvana Meira, Juliana Cobre

**Affiliations:** ^1^FACET, Universidade Federal da Grande Dourados, Brazil; ^2^ICMC, Universidade de São Paulo, Brazil; ^3^DEs, Universidade Federal de São, Carlos, Brazil

## Abstract

A common interest in gene expression data analysis is to identify from a large pool of candidate genes the genes that present significant changes in expression levels between a treatment and a control biological condition. Usually, it is done using a statistic value and a cutoff value that are used to separate the genes differentially and nondifferentially expressed. In this paper, we propose a Bayesian approach to identify genes differentially expressed calculating sequentially credibility intervals from predictive densities which are constructed using the sampled mean treatment effect from all genes in study excluding the treatment effect of genes previously identified with statistical evidence for difference. We compare our Bayesian approach with the standard ones based on the use of the *t*-test and modified *t*-tests via a simulation study, using small sample sizes which are common in gene expression data analysis. Results obtained report evidence that the proposed approach performs better than standard ones, especially for cases with mean differences and increases in treatment variance in relation to control variance. We also apply the methodologies to a well-known publicly available data set on *Escherichia coli* bacterium.

## 1. Introduction

The DNA array technology is capable of providing gene expression levels measurements for thousands of genes simultaneously under different biological experimental conditions. In these experiments, total RNA is reverse-transcribed to create either radioactive or fluorescent-labeled cDNA which is hybridized with a large DNA library of gene fragments attached to a glass or membrane support [1]. After this, a scanner of high resolution is used to obtain the color intensity from each spot. So, the color intensities are normalized in order to obtain the expression level of genes. For further discussion and additional references on DNA array technology see [2–9].

Obtaining the expression levels, a common objective is to identify genes that present significant changes in gene expression levels between treatment and control experimental condition. The identification of these genes is important because it may bring to light new biological discoveries, such as which genes may be involved in the origin and/or evolution of the same disease of genetic origin or which genes react to a drug stimulus. Thus, we aim to establish the use of these experiments as tools in medicine [10].

As the observed expression levels incorporate different sources of variability present in the process of obtaining fluorescent intensity measurements [11], statistical methods are important to identify the genes differentially expressed. One of the first approaches proposed to identify genes differentially expressed was the fold-change approach [2, 3]. In this approach, a gene is considered differentially expressed if the average of the logarithm of the observed expression levels in treatment and control varies more than a cutoff point, *R*_*c*_, which is previously prefixed. This approach however is not adequate to yield good results, once a cutoff value *R*_*c*_ may have different significance for different observed expression levels. Besides, this approach does not consider the variability of the observed expression levels from treatment and control.

Another method commonly used for gene expression data analysis is the so-called two-sample *t*-test (TT) for the log transformed data [1, 8]. The problem with the application of TT to this kind of data is the usual small size of treatment and control samples in genetic studies, which may lead to underestimated variances and small power of test. To avoid such limitations, some TT modifications were proposed, such as the Cyber-*t* (CT) proposed by [1] and the Bayesian *t*-test (BTT) proposed by [12]. Basically, the main idea is to consider modifications of the standard error estimate of the two-sample difference present in the denominator of the standard *t*-statistics.

In this paper, we propose a Bayesian approach to identify genes differentially expressed by calculating sequentially credibility intervals from predictive densities which are constructed using all treatment effects excluding the treatment effect of genes previously identified with statistical evidence for difference. This procedure avoids the small sample size problem, usual in gene expression data analysis, and allows us to use the normality assumption for observed data [9].

In order to verify the performance of the proposed approach and compare it with the conventional ones based on the use of the *t*-test and modified *t*-tests, we present a simulation study. The comparison is done in terms of the true positive rate, false positive rate, and true discovery rate. Results obtained report evidence that our proposed approach performs better than *t*-test and modified *t*-tests, especially for cases with mean differences and increases in treatment variance in relation to control variance. We also apply the methods to a real dataset, obtained from the experiment carried through with *Escherichia coli* bacterium, described in [5].

The paper is organized as follows. In Section 2, we develop our Bayesian approach constructing the predictive density and describing our criteria to identify the genes differentially expressed. In Section 3, the method is compared with the *t*-test and modified *t*-tests using simulated datasets and a real dataset. In Section 4, we conclude the paper with final remarks on the proposed method.

## 2. Predictive Modeling for Gene Expression

 Consider a DNA array experiment with *n* genes and two experimental conditions which we name by control (*c*) and treatment (*t*). Suppose that control and treatment are replicated *n*_*c*_ and *n*_*t*_ times, respectively. Denote by *x*_*ig*_*h*__ the *i*th observed expression level (or its logarithm) for gene *g* in experimental condition *h*, *h* ∈ {*c*, *t*}, and *g* = 1,…, *n*. Let **x**_*g*_*h*__ = {*x*_1*g*_*h*__,…, *x*_*n*_*h*_*g*_*h*__} be realizations of independent random variables **X**_*g*_*h*__ = {*X*_1*g*_*h*__,…, *X*_*n*_*h*_*g*_*h*__}, for *g* = 1,…, *n* and *h* ∈ {*c*, *t*}.

Consider that

(1) $$\begin{array}{c} Y_{g}=\frac{1}{n_{t}}\overset{n_{t}}{\underset{i=1}{\sum }}X_{ig_{t}}-\frac{1}{n_{c}}\overset{n_{c}}{\underset{i=1}{\sum }}X_{ig_{c}} \end{array}$$

is the sampled mean treatment effect for gene *g*, *g* = 1,…, *n*, and **Y** = {*Y*_1_,…, *Y*_*n*_} is the set of all sampled mean treatment effects.

Thus, considering **Y**, we can determine the predictive density for a new observation *Y*_*n*+1_, given **Y**, and build a 100(1 − *α*)% credibility interval for *Y*_*n*+1_. In order to develop our idea and as often found in gene expression data analysis [1, 8, 9, 11–13], we assume that **Y** is an independent sample generated from a normal distribution with mean *μ* and variance *σ* ^2^,

(2) $$\begin{array}{c} Y_{1},Y_{2},\ldots ,Y_{n}\sim 𝒩\left(\mu ,\sigma^{2}\right). \end{array}$$

The likelihood function is given by

(3) $$\begin{array}{c} L\left(\mu ,\sigma^{2}\right)\propto {\left(\sigma^{2}\right)}^{-n/2}\exp\left\{-\frac{n{\left(\bar{y}-\mu \right)}^{2}+\left(n-1\right)s^{2}}{2\sigma^{2}}\right\}, \end{array}$$

where $\bar{y}=(1/n)\sum_{g=1}^{n}y_{g}$ and $s^{2}=(1/(n-1))\sum_{g=1}^{n}{\left(y_{g}-\bar{y}\right)}^{2}$ are the sample mean and variance of **y**, respectively.

Since parameters *μ* and *σ* ^2^ have a direct interpretation in the context of the gene expression data analysis, so we may express expert opinions in terms of prior distributions for parameters. In order to explore the fully conjugation, consider that joint prior distribution for parameters *μ* and *σ* ^2^ is given by

(4) $$\begin{array}{c} \mu \;|\;\sigma^{2}\sim 𝒩\left(\mu_{0},\frac{\sigma^{2}}{\lambda }\right),\;\;\sigma^{2}\sim ℐ𝒢\left(\frac{\tau }{2},\frac{\beta }{2}\right), \end{array}$$

where *μ*_0_, *λ*, *τ*, and *β* are known hyperparameters, and *ℐ𝒢*(·) represent the inverse gamma distribution with mean (*β*/2)/(*τ*/2) − 1.

Updating the prior distributions in (4) via likelihood function in (3), the joint posterior distribution for (*μ*, *σ* ^2^) is given by

(5) $$\begin{array}{c} \mu ,\sigma^{2}\;|\;y\sim 𝒩\left(\mu^{*},\frac{\sigma^{2}}{\lambda +n}\right)ℐ𝒢\left(\frac{\tau^{*}}{2},\frac{\beta^{*}}{2}\right), \end{array}$$

where $\mu^{*}=(n/(n+\lambda )\bar{y})+(\lambda /(n+\lambda ))\mu_{0}$, τ* = *τ* + *n* + 1, and $\beta^{*}=\beta +(n-1)\cdot s^{2}+n\lambda {\left(\bar{y}-\mu_{0}\right)}^{2}/(n+\lambda )$, for *g* = 1,…, *n*.

Considering now that *Y*_*n*+1_ is a new observation, independent from **Y**, the predictive distribution of *Y*_*n*+1_ | **y** is given by

(6) $$\begin{array}{c} Y_{n+1}\;|\;y\sim t_{\tau^{*}}\left(\mu^{*},\frac{\beta^{*}\left(n+\lambda +1\right)}{\tau^{*}\left(n+\lambda \right)}\right), \end{array}$$

where *t*_τ*_ represents the Student's *t*-distribution with location parameter μ*, scale β*(*n* + *λ* + 1)/τ*(*n* + *λ*), and τ* degrees of freedom.

From (6), the variance of *Y*_*n*+1_ | **y** is given by

(7) $$\begin{array}{c} \mathrm{Var}\left(Y_{n+1}\;|\;y\right)=\left(\frac{\tau^{*}}{\tau^{*}-2}\right)\cdot \left(\frac{\beta^{*}\left(n+\lambda +1\right)}{\tau^{*}\left(n+\lambda \right)}\right), \end{array}$$

and a 100(1 − *α*)% credibility interval for *Y*_*n*+1_ | **y** is given by

(8) $$\begin{array}{cc} I_{\left(1-\alpha \right)}\left(Y_{n+1}\;|\;y\right) & =(\mu^{*}-t_{1-\alpha /2,\tau^{*}}\sqrt{\mathrm{Var}\left(Y_{n+1}\;|\;y\right)},\mu^{*}\\ & \;\;+t_{1-\alpha /2,\tau^{*}}\sqrt{\mathrm{Var}\left(Y_{n+1}\;|\;y\right)}), \end{array}$$

where *t*_1−*α*/2,τ*_ denotes the quantile 1 − *α*/2 of the standard *t*-student distribution with τ* degrees of freedom.

### 2.1. Predictive Approach Criterion

Let **y** ^ord^ = {*y*_(1)_, *y*_(2)_,…, *y*_(*n*)_}, the set **y** in increasing numerical order, *y*_(1)_ < *y*_(2)_ < ⋯<*y*_(*n*)_. Assuming that *y*_(*g*)_ is a future observation in relation to the set composite by all observed treatment effect except the *g*th treatment effect, **y**_(−*g*)_^ord^ = {*y*_(1)_,…, *y*_(*g*−1)_, *y*_(*g*+1)_,…, *y*_(*n*)_}, so the distribution of *Y*_(*g*)_ is given by (6) with posterior parameter calculated using **y**_(−*g*)_^ord^ and *I*_1−*α*_(*Y*_(*g*)_ | **y**_(−*g*)_^ord^) is a 100(1 − *α*)% credibility interval for *Y*_(*g*)_, *g* = 1,2,…, *n*.

In order to identify the genes differentially expressed, we fix *E*(*μ* | **y**_(−*g*)_^ord^) = μ* = 0 and set up $I_{\left(g\right)}^{\text{threshold}}=t_{1-\alpha /2,\tau^{*}}\sqrt{\mathrm{Var}\left(\left\{y_{\left(-g\right)}^{\text{ord}}\right\}\setminus \{y^{\text{dif}}\}\right)}$ as a threshold, where {**y** ^dif^} is the set that will be composite by treatment effect from genes identified with evidences for difference, and Var⁡({**y**_(−*g*)_^ord^}∖{**y** ^dif^}) is calculated according to (7) for the set {**y**_(−*g*)_^ord^} excluding the set {**y** ^dif^}. For *g* = 1,…, *n*, the identification of the genes differentially expressed is given by the following steps:

- Calculate *I*_(*g*)_^threshold^;
- if |*y*_(*g*)_ | ≤ *I*_(*g*)_^threshold^, then gene *g* does not presents statistical evidence for differences;
- if |*y*_(*g*)_ | > *I*_(*g*)_^threshold^, then gene *g* present statistical evidence for differences. Do **y** ^dif^ = **y** ^dif^ ∪ *y*_(*g*)_.

## 3. Data Analysis

 In this section, we illustrate the predictive approach (PA) applied to artificial and real datasets. The real data set was extracted from the site (http://www.jbc.org/) and refers to an experiment realized with *Escherichia Coli* bacterium using nylon membranes, described by [5].

Moreover, we compare the PA results with the results obtained by considering three well-known methods to identify differentially expressed genes: the two-sample *t*-test (TT) and Cyber-*t* test (CT) proposed by [1] and with the Bayesian *t*-test (BTT) proposed by [12].

In the TT, the hypothesis test is based on the statistics

(9) $$\begin{array}{c} t_{g}=\frac{{\bar{x}}_{g_{t}}-{\bar{x}}_{g_{c}}}{\sqrt{s_{g_{t}}^{2}/n_{t}+s_{g_{c}}^{2}/n_{c}}}, \end{array}$$

which follows a Student's *t*-distribution with *df* = [*s*_*g*_*c*__^2^/*n*_*c*_ + *s*_*g*_*t*__^2^/*n*_*t*_]^2^/[(*s*_*g*_*c*__^2^/*n*_*c*_)^2^/(*n*_*c*_ − 1) + (*s*_*g*_*t*__^2^/*n*_*t*_)^2^/(*n*_*t*_ − 1)], degrees of freedom, where ${\bar{x}}_{{\text{g}}_{h}}$ and *s*_*g*_*h*__^2^ are the sample mean and variance for gene *g* in experimental condition *h* = {*c*, *t*}. Fixing a significance level *α*, if |*t*_*g*_| is greater than a threshold *t*_1−*α*/2,*df*_ (quantile 1 − *α*/2 of Student's *t* distribution with *df* degrees of freedom), then the test conclude for difference of expression levels.

Reference [1] proposed a two-sample *t*-test replacing the denominator of (1) by a pooled variance estimated via a Bayesian approach. So, the authors implement the Cyber-*t* software using the statistics

(10) $$\begin{array}{c} t_{g}=\frac{{\bar{x}}_{g_{t}}-{\bar{x}}_{g_{c}}}{\sqrt{{\tilde{\sigma }}_{g_{t}}^{2}/n_{g_{t}}+{\tilde{\sigma }}_{g_{c}}^{2}/n_{g_{c}}}} \end{array}$$

and the degrees of freedom *df* = *ν*_0_ + *n*_*g*_*c*__ + *n*_*g*_*t*__ − 2, where ${\tilde{\sigma }}_{g_{h}}^{2}=\nu_{0}\sigma_{0}^{2}+(n_{h}-1)s_{g_{h}}^{2}/(\nu_{0}+n_{h}-2)$, for *h* ∈ {*c*, *t*}, where *ν*_0_ and *σ*_0_^2^ are hyperparameters. The authors assume that *k* > 2 points are needed to properly estimate the standard deviation and keep *n*_*g*_ + *ν*_0_ = *k*, where *n*_*g*_ = *n*_*g*_*c*__ + *n*_*g*_*t*__. They suggest to fix *k* = 10 and so *ν*_0_ = 10 − *n*_*g*_. To fix a value for *σ*_0_^2^, the authors say “one could use the standard deviation of the entire set of observations or, depending on the situation, of particularly categories of genes.” Using only the information from observations of the gene *g*, we fix *σ*_0_^2^ = ((*n*_*g*_ − 1)/*n*_*g*_)*s*_*g*_^2^, as suggested by [1], where *s*_*g*_^2^ is the sample variance of the set **x**_*g*_ = {**x**_*g*_*c*__, **x**_*g*_*t*__}.

Based on [1, 12], develop a Bayesian approach and show that

(11) $$\begin{array}{c} \frac{\Delta \mu -\Delta \bar{x}}{\sigma_{n}\sqrt{1/n_{g_{t}}+1/n_{g_{c}}}}|x_{g_{c}},\;\;x_{g_{t}}\sim t_{\nu_{n}}, \end{array}$$

where $\Delta \bar{x}={\bar{x}}_{g_{t}}-{\bar{x}}_{g_{c}},\;\nu_{n}\sigma_{n}^{2}=\nu_{0}\sigma_{0}^{2}+(n_{g_{c}}-1)s_{g_{c}}^{2}+(n_{g_{t}}-1)s_{g_{t}}^{2}$, *ν*_*n*_ = *ν*_0_ + *n*_*g*_*c*__ + *n*_*g*_*t*__ − 2, and *t*_*ν*_*n*__ represent the Student's *t* distribution with *ν*_*n*_ degrees of freedom, for *g* = 1,…, *n*. As suggested by authors, we fix *ν*_0_ = *n*_*g*_ and *σ*_0_^2^ = *s*_*g*_^2^.

### 3.1. Artificial Data

To generate the artificial data sets, we consider that observations from control group are generated from a normal distribution with mean *μ*_*c*_ and variance *σ*_*c*_^2^, *X*_*ig*_*c*__ ~ *𝒩*(*μ*_*c*_, *σ*_*c*_^2^), for *i* = 1,…, *n*_*c*_ and *g* = 1,…, *n*. We fix *μ*_*g*_*c*__ = −14 and *σ*_*g*_*c*__^2^ = 0.8. These values are the average of the observed mean and variance of the expression levels (log transformed) from control group of the *Escherichia coli* bacterium dataset. We fix *n* = 1.000, and the sample sizes *n*_*c*_ and *n*_*t*_ were fixed at 4 and 8.

To generate the observations from treatment group, we follow the steps:

- From index {1,…, *n*}, we choose randomly *p*% of these index to indicate the cases generated with difference, *p* ∈ {5,10,20};
- if the index *g* ∈ {1,…, *n*} is chosen, then we consider an indicator variable *𝕀*_*g*_ = 1 and generate *X*_*ig*_*t*__ ~ *𝒩*(*μ*_*t*_, *σ*_*t*_^2^), for *i* = 1,…, *n*_*t*_. In order to verify how the method behaves when (*μ*_*t*_, *σ*_*t*_^2^) moves away from (*μ*_*c*_, *σ*_*c*_^2^), we simulate its values using

(12) $$\begin{array}{c} \mu_{g_{t}}=\mu_{g_{c}}\pm \delta \sigma_{c},\;\;\sigma_{t}=\gamma \sigma_{c}, \end{array}$$

for *δ* = {0,0.25,0.50,0.75,1,  1.25,1.50,1.75,2} and *γ* = {1,2, 3}. The differential cases represent the situation overexpressed, defined by signal + in expression *μ*_*t*_, and under expressed, defined by the signal − in expression of *μ*_*t*_. We use *p* = *p*_over_ + *p*_under_, for *p*_over_ = {3,7, 5} and *p*_under_ = {2,3, 15}, respectively. For example, *p* = 5 is composite by *p*_over_ = 3 plus *p*_under_ = 2

(iii) if the index *g* ∈ {1,…, *n*} is not chosen, then set up *𝕀*_*g*_ = 0 and generate *X*_*ig*_*t*__ ~ *𝒩*(*μ*_*c*_, *σ*_*c*_^2^), for *i* = 1,…, *n*_*c*_.

For PA application, we fix the hyperparameters in order to have weak informative priors. We set up (i) *τ* and *β* in a way that *E*[*σ* ^2^] = (*β*/2)/((*τ*/2) − 1) = *R* ^2^, where *R* = max⁡(**y**) − min⁡(**y**) is the length of the interval of variation of the observed data **y**. Thus, we obtain *β* = (*τ* − 2) · *R* ^2^. So, we fix *τ* = 3, *μ*_0_ = 0, and *λ* = 10^−2^. We also set up *α* = 0.05 to calculate the credibility intervals and for the *t*-tests.

To record the cases identified with difference by PA, we consider an indicator variable *𝕀*_*g*_^PA^ = 1 for cases, so that |*y*_(*g*)_ | > *I*_(*g*)_^threshold^. Otherwise, *𝕀*_*g*_^PA^ = 0. Analogously, for TT, CT, and BTT, we consider *𝕀*_*g*_^method^ = 1 (method = {TT, CT, BTT}) for cases with *P*value_*g*_ < 0.05 and *𝕀*_*g*_^method^ = 0, otherwise.

In order to compare the performance of methods, we calculate the true positive rate given by

(13) $$\begin{array}{c} P_{\text{method}}=\frac{\sum_{g=1}^{n}{𝕀}_{g}\cdot {𝕀}_{g}^{\text{method}}}{\sum_{g=1}^{n}{𝕀}_{g}}, \end{array}$$

where method = {PA, TT, CT, BTT}.

We calculate the true positive rate for *S* = 100 different datasets generated according to steps (i) to (iii) described above, and we present the results using the mean of the true positive rate, that is given by ${\bar{P}}_{\text{method}}=\sum_{s=1}^{S}P_{\text{method}}^{\left(s\right)}/S$, where *P*_method_^(*s*)^ is the true positive rate calculated for *s*th generated dataset for method = {PA, TT, CT, BTT}.

Tables 1, 2, and 3 present the ${\bar{P}}_{\text{method}}$ value for *n*_*c*_ = *n*_*t*_ = 4 and Tables 4, 5, and 6 present the ${\bar{P}}_{\text{method}}$ value for *n*_*c*_ = *n*_*t*_ = 8, for method = {PA, TT, CT, BTT}.

As we move from the left to the right side of the tables, in each line, we have the distances between control and treatment means, which are increasing. As we move from top to down in columns of the tables, we have the distance between the treatment and control variances, which are increasing. Increasing the sample sizes from 4 to 8, all methods increase its performance.

For *n*_*c*_ = 4 and all values of *δ* and *γ* used, the PA present better performance than TT, CT, and BTT. Moving away the treatment distribution from the control distribution (increasing *δ* and *γ*), the true positive rate obtained by PA is greater than *t*-tests.

For *n*_*c*_ = 8 and *γ* = 1 fixed the TT, CT, and BTT present better performance than PA for the same values of *δ* used. For example, for *p* = 5 and *δ* = {1.5,1.75,2} the *t* tests present greater true positive rate than PA. But increasing the value of *γ*, *γ* = {2,3}, the PA presents greater true positive rate.

Besides, note that TT, CT, and BTT present similar results with a slight advantage for BTT, that is, greater true positive rate than TT and CT. Also note that true positive rate obtained by CT is smaller than TT for all cases simulated. It happens because we use only the information from observations from gene *g* to fix the hyperparameter *σ*_0_^2^. In order to obtain better results, [1, 12] suggest to fix *σ*_0_ as the standard deviation estimated by pooling together all the neighboring genes contained in a window of size *w*. But, the authors do not discuss how to define a good value *w* to lead to satisfactory results.

We also compare the performance of the methods using the mean of the false positive rate and the mean of the true discovery rate. The mean of the false positive rates is presented in Tables 7–9 and in Tables 10–12. All methods present a small false positive rate.

The mean of the true discovery rates is presented in Tables 13–15 and in Tables 16–18. The PA presents greater true discovery rate than *t*-tests for all values of *δ* and *γ* used. Besides, note that increasing the value of *δ* and *γ*, the true discovery rate increases in both directions for PA. But the same does not happen with the *t*-tests, in which the proportion of identification increases only as the value of *δ* increases, that is, when the mean of the treatment distribution moves away from the mean of the control distribution. Increasing the variance of treatment (increasing the value of *γ*), the *t*-tests present a reduction of its performance, in opposite to PA which presents an improvement in its performance.

Results show a better performance of the PA in relation to TT, CT, and BTT, specially, when the difference refers to variance of the variable involved. From the biological practical point of view, it shows us that PA may identify with differences genes which are not identified by TT, CT, and BTT, specially, genes with differences in means and variances.

### 3.2. Escherichia coli Data

**Figure 1:**
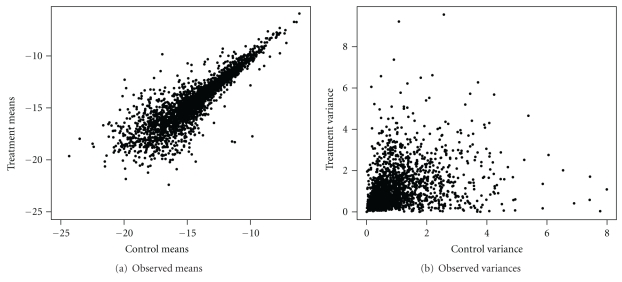
Treatment and control observed means and variances.

In this section consider the gene expression data set on *Escherichia coli* bacterium, composed by *n* = 4290 genes [5].  Figure 1 shows the treatment and control observed means and variances for all genes of this dataset.

**Figure 2:**
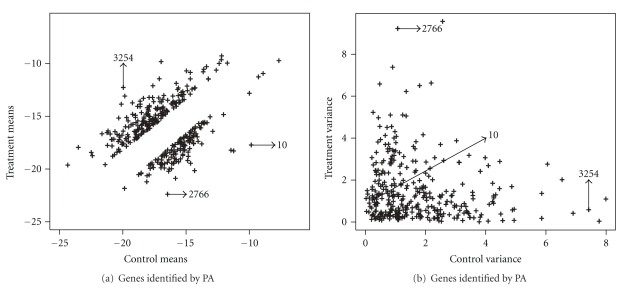
Treatment and control observed means and variances and genes identified with evidence for difference by PA.

**Figure 3:**
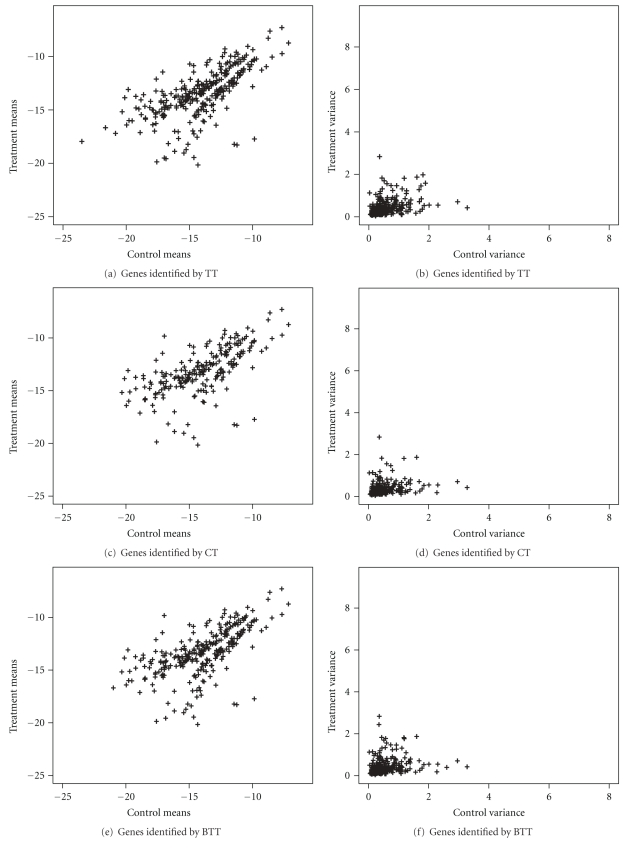
Treatment and control observed means and variances and genes identified with evidence for difference by TT, CT, and BTT.

Results for PA are presented in Figure 2. Results for TT, CT, and BTT are presented in Figure 3. These figures show the observed treatment and control means and variances of genes identified with evidence for difference by considering PA, TT, CT, and BTT, respectively. The PA identifies 340 genes with evidences for difference, while TT identifies 222, CT 219, and BTT 288 genes.

Note that genes with means well apart are better identified by PA than by the other methods. Moreover, genes with mean and variances well apart are identified by PA and not identified by TT, CT, and BTT, as can be noted in Figure 2. Examples are genes 2766 (b1326(f262)) and 3254 (dbpA) that are highlighted in Figures 2(a) and 2(b). Genes with means well apart and similar variances are however identified by TT, CT, and BTT. An example is the gene 10 (hdeB) that is highlighted in Figures 2(a) and 2(b). One possible reason for this is the low performance of TT, CT and BTT in situations with differences in means and variances, as observed in the artificial data sets. Besides, note that PA is capable to identify differentially expressed genes which are not identified by TT, CT, and BTT, specially, genes with differences in means and variances.

## 4. Discussion

 Identifying genes with difference, in what concerns gene expression, may help biologists to study and understand some function of genes and infer possible relationships among genes and proteins. In this paper we propose a Bayesian approach to identify differentially expressed genes based on predictive density.

In order to verify the performance of the PA approach and compare it with TT, CT, and BTT, we considered artificial and real datasets. Results show a better performance of PA in relation to the *t*-tests in identifying difference, mainly, in presence of different variances. The main advantage of the proposed method is that it is easy to use like a usual two-sample *t*-test but presents better performance in situations with small sample size.

The biological interest in this fact is that PA may bring to light genes that are not identified when we use only the TT or the modified *t*-test ones. Moreover, the PA can be easily implemented in usual softwares such as the software *R* (the Comprehensive *R* Archive Network, http://cran.r-project.org/). The source code used for the data analysis was implemented in software *R* and can be obtained by emailing the authors.

According to [1], gene expression data can be analyzed in at least three levels of increasing complexity. In the first level, each gene is analyzed separately, where the objective is to verify whether the observed expression in treatment experimental condition is significantly different from observed expression in control experimental condition. In the second level, clusters of genes are analyzed in terms of common functionalities and interactions. In the third level, the objective is to infer and understand the relationship among genes. As it should be clear by now, in this paper, we focus on the first level of analysis. However, as future work we intent to present the adjustment of the proposed method to control the false discovery rate for multiple testing hypotheses, when thousands of hypotheses are realized simultaneously, as proposed by [14], as well as to make a systematic comparison with his methodology. Besides, we also will development a multivariate approach in order to consider dependence among genes.

## Figures and Tables

**Table 1:** True positive rate, *n*_*c*_ = *n*_*t*_ = 4, *p* = 5% (3% over and 2% under).

*γ*	Method	*δ*								
		0	0.25	0.50	0.75	1	1.25	1.5	1.75	2
1	PA	0.067	0.072	0.120	0.189	0.308	0.410	0.532	0.653	0.738
	TT	0.040	0.046	0.072	0.122	0.198	0.290	0.386	0.507	0.601
	CT	0.031	0.037	0.056	0.092	0.160	0.243	0.323	0.442	0.533
	BTT	0.041	0.046	0.073	0.122	0.200	0.295	0.388	0.507	0.604

2	PA	0.220	0.229	0.258	0.308	0.363	0.425	0.492	0.560	0.630
	TT	0.050	0.049	0.063	0.089	0.107	0.136	0.182	0.221	0.277
	CT	0.038	0.042	0.050	0.073	0.092	0.118	0.155	0.199	0.250
	BTT	0.053	0.054	0.064	0.092	0.114	0.145	0.194	0.239	0.299

3	PA	0.357	0.370	0.367	0.397	0.424	0.454	0.486	0.530	0.584
	TT	0.049	0.057	0.059	0.067	0.081	0.102	0.112	0.145	0.170
	CT	0.045	0.051	0.050	0.058	0.074	0.094	0.106	0.138	0.162
	BTT	0.060	0.063	0.068	0.077	0.094	0.116	0.133	0.164	0.195

**Table 2:** True positive rate, *n*_*c*_ = *n*_*t*_ = 4, *p* = 10% (7% over and 3% under).

*γ*	Method	*δ*								
		0	0.25	0.50	0.75	1	1.25	1.5	1.75	2
1	PA	0.063	0.076	0.110	0.181	0.257	0.359	0.447	0.538	0.631
	TT	0.043	0.054	0.074	0.121	0.191	0.286	0.379	0.493	0.613
	CT	0.032	0.040	0.054	0.096	0.154	0.233	0.321	0.426	0.545
	BTT	0.043	0.055	0.074	0.123	0.193	0.286	0.384	0.497	0.616

2	PA	0.208	0.211	0.237	0.275	0.316	0.367	0.434	0.497	0.556
	TT	0.045	0.052	0.064	0.085	0.103	0.141	0.184	0.233	0.286
	CT	0.036	0.042	0.050	0.070	0.086	0.120	0.156	0.205	0.259
	BTT	0.048	0.054	0.067	0.090	0.108	0.150	0.195	0.253	0.312

3	PA	0.317	0.314	0.337	0.346	0.365	0.407	0.434	0.473	0.510
	TT	0.049	0.058	0.056	0.071	0.080	0.099	0.118	0.144	0.173
	CT	0.042	0.051	0.049	0.060	0.072	0.090	0.110	0.134	0.165
	BTT	0.056	0.065	0.065	0.080	0.091	0.111	0.136	0.166	0.200

**Table 3:** True positive rate, *n*_*c*_ = *n*_*t*_ = 4, *p* = 20% (5% over and 15% under).

*γ*	Method	*δ*								
		0	0.25	0.50	0.75	1	1.25	1.5	1.75	2
1	PA	0.066	0.077	0.117	0.176	0.241	0.322	0.411	0.510	0.610
	TT	0.043	0.049	0.079	0.123	0.197	0.285	0.384	0.494	0.606
	CT	0.031	0.035	0.060	0.098	0.157	0.232	0.322	0.430	0.540
	BTT	0.042	0.049	0.080	0.124	0.197	0.287	0.387	0.501	0.613

2	PA	0.179	0.187	0.223	0.260	0.313	0.369	0.425	0.487	0.545
	TT	0.049	0.051	0.063	0.083	0.103	0.142	0.184	0.236	0.286
	CT	0.039	0.040	0.051	0.069	0.084	0.122	0.160	0.208	0.254
	BTT	0.051	0.055	0.067	0.088	0.110	0.153	0.197	0.253	0.307

3	PA	0.274	0.276	0.303	0.328	0.356	0.383	0.428	0.464	0.511
	TT	0.052	0.056	0.063	0.070	0.080	0.098	0.122	0.142	0.175
	CT	0.045	0.049	0.055	0.063	0.073	0.090	0.113	0.135	0.167
	BTT	0.059	0.064	0.071	0.079	0.093	0.113	0.138	0.165	0.201

**Table 4:** True positive rate, *n*_*c*_ = *n*_*t*_ = 8, *p* = 5% (3% over and 2% under).

*γ*	Method	*δ*								
		0	0.25	0.50	0.75	1	1.25	1.5	1.75	2
1	PA	0.061	0.084	0.179	0.323	0.489	0.636	0.787	0.879	0.939
	TT	0.047	0.075	0.145	0.282	0.460	0.630	0.798	0.899	0.957
	CT	0.038	0.057	0.120	0.242	0.415	0.580	0.765	0.873	0.944
	BTT	0.047	0.073	0.144	0.283	0.459	0.630	0.798	0.897	0.958

2	PA	0.218	0.237	0.304	0.379	0.477	0.583	0.663	0.751	0.819
	TT	0.051	0.059	0.088	0.136	0.211	0.301	0.403	0.510	0.621
	CT	0.043	0.051	0.079	0.123	0.188	0.274	0.377	0.487	0.597
	BTT	0.053	0.062	0.094	0.143	0.219	0.314	0.422	0.532	0.640

3	PA	0.352	0.365	0.387	0.433	0.481	0.547	0.607	0.671	0.731
	TT	0.048	0.059	0.072	0.091	0.129	0.174	0.224	0.290	0.361
	CT	0.043	0.053	0.066	0.086	0.124	0.166	0.218	0.280	0.354
	BTT	0.055	0.068	0.077	0.104	0.145	0.192	0.247	0.318	0.393

**Table 5:** True positive rate, *n*_*c*_ = *n*_*t*_ = 8, *p* = 10% (7% over and 3% under).

*γ*	Method	*δ*								
		0	0.25	0.50	0.75	1	1.25	1.5	1.75	2
1	PA	0.067	0.088	0.168	0.287	0.407	0.536	0.664	0.773	0.858
	TT	0.052	0.071	0.146	0.282	0.443	0.626	0.785	0.894	0.957
	CT	0.041	0.056	0.125	0.248	0.399	0.581	0.751	0.870	0.945
	BTT	0.051	0.070	0.146	0.281	0.443	0.625	0.785	0.894	0.957

2	PA	0.204	0.224	0.267	0.332	0.416	0.494	0.579	0.648	0.728
	TT	0.050	0.059	0.091	0.135	0.214	0.304	0.402	0.508	0.628
	CT	0.042	0.050	0.080	0.122	0.193	0.280	0.378	0.485	0.604
	BTT	0.052	0.063	0.095	0.143	0.223	0.316	0.419	0.525	0.647

3	PA	0.332	0.325	0.340	0.383	0.418	0.478	0.531	0.580	0.634
	TT	0.050	0.056	0.071	0.093	0.127	0.171	0.223	0.285	0.356
	CT	0.046	0.052	0.066	0.088	0.121	0.163	0.215	0.277	0.348
	BTT	0.057	0.063	0.079	0.103	0.142	0.193	0.246	0.313	0.387

**Table 6:** True positive rate, *n*_*c*_ = *n*_*t*_ = 8, *p* = 20% (5 over and 15 under).

*γ*	Method	*δ*								
		0	0.25	0.50	0.75	1	1.25	1.5	1.75	2
1	PA	0.066	0.092	0.162	0.263	0.381	0.513	0.658	0.768	0.864
	TT	0.052	0.073	0.150	0.278	0.454	0.632	0.790	0.895	0.958
	CT	0.040	0.060	0.125	0.243	0.407	0.589	0.754	0.872	0.945
	BTT	0.051	0.072	0.149	0.277	0.453	0.632	0.790	0.896	0.957

2	PA	0.177	0.206	0.253	0.318	0.408	0.499	0.575	0.652	0.725
	TT	0.051	0.061	0.088	0.139	0.212	0.303	0.404	0.507	0.627
	CT	0.042	0.054	0.078	0.124	0.194	0.281	0.380	0.482	0.605
	BTT	0.054	0.064	0.093	0.145	0.222	0.317	0.422	0.528	0.647

3	PA	0.271	0.294	0.322	0.370	0.417	0.468	0.526	0.587	0.630
	TT	0.052	0.057	0.067	0.095	0.125	0.175	0.227	0.282	0.350
	CT	0.049	0.053	0.062	0.091	0.119	0.169	0.220	0.275	0.343
	BTT	0.060	0.066	0.077	0.108	0.140	0.194	0.252	0.310	0.383

**Table 7:** False positive rate, *n*_*c*_ = *n*_*t*_ = 4, *p* = 5% (3 over and 2 under).

*γ*	Method	*δ*								
		0	0.25	0.50	0.75	1	1.25	1.5	1.75	2
1	PA	0.062	0.062	0.058	0.054	0.050	0.045	0.039	0.033	0.028
	TT	0.040	0.041	0.042	0.041	0.040	0.042	0.042	0.041	0.040
	CT	0.030	0.030	0.031	0.029	0.029	0.030	0.031	0.030	0.029
	BTT	0.041	0.041	0.042	0.041	0.040	0.042	0.042	0.041	0.040

2	PA	0.051	0.051	0.050	0.045	0.041	0.037	0.031	0.027	0.023
	TT	0.041	0.042	0.042	0.041	0.041	0.041	0.041	0.040	0.041
	CT	0.030	0.031	0.030	0.030	0.029	0.030	0.030	0.029	0.030
	BTT	0.041	0.042	0.042	0.041	0.041	0.042	0.042	0.040	0.041

3	PA	0.040	0.037	0.038	0.033	0.030	0.025	0.023	0.018	0.016
	TT	0.040	0.041	0.040	0.041	0.043	0.040	0.041	0.042	0.040
	CT	0.029	0.030	0.029	0.030	0.031	0.029	0.030	0.031	0.030
	BTT	0.040	0.041	0.040	0.041	0.043	0.040	0.041	0.042	0.041

**Table 8:** False positive rate, *n*_*c*_ = *n*_*t*_ = 4, *p* = 10% (7 over and 3 under).

*γ*	Method	*δ*								
		0	0.25	0.50	0.75	1	1.25	1.5	1.75	2
1	PA	0.061	0.060	0.056	0.048	0.039	0.031	0.023	0.017	0.012
	TT	0.040	0.040	0.041	0.042	0.042	0.041	0.041	0.040	0.041
	CT	0.029	0.030	0.030	0.030	0.030	0.030	0.030	0.030	0.030
	BTT	0.040	0.041	0.041	0.042	0.041	0.041	0.041	0.040	0.041

2	PA	0.046	0.044	0.039	0.033	0.027	0.021	0.017	0.012	0.009
	TT	0.041	0.040	0.042	0.042	0.040	0.041	0.041	0.041	0.041
	CT	0.030	0.029	0.031	0.030	0.029	0.030	0.030	0.030	0.030
	BTT	0.041	0.040	0.041	0.041	0.040	0.041	0.041	0.041	0.041

3	PA	0.029	0.026	0.023	0.020	0.016	0.013	0.010	0.007	0.005
	TT	0.041	0.042	0.040	0.042	0.041	0.041	0.040	0.041	0.041
	CT	0.030	0.030	0.029	0.030	0.029	0.031	0.029	0.030	0.030
	BTT	0.041	0.042	0.040	0.042	0.041	0.042	0.041	0.041	0.042

**Table 9:** False positive rate, *n*_*c*_ = *n*_*t*_ = 4, *p* = 20% (5 over and 15 under).

*γ*	Method	*δ*								
		0	0.25	0.50	0.75	1	1.25	1.5	1.75	2
1	PA	0.062	0.062	0.055	0.046	0.036	0.027	0.020	0.014	0.011
	TT	0.040	0.040	0.041	0.041	0.041	0.041	0.042	0.042	0.041
	CT	0.030	0.029	0.029	0.029	0.030	0.030	0.030	0.030	0.030
	BTT	0.040	0.041	0.040	0.041	0.041	0.041	0.041	0.042	0.042

2	PA	0.034	0.035	0.034	0.029	0.027	0.020	0.016	0.011	0.008
	TT	0.041	0.041	0.042	0.041	0.041	0.040	0.040	0.042	0.041
	CT	0.030	0.030	0.031	0.029	0.029	0.029	0.029	0.030	0.029
	BTT	0.041	0.041	0.042	0.041	0.041	0.040	0.040	0.042	0.041

3	PA	0.016	0.016	0.015	0.015	0.014	0.011	0.010	0.007	0.005
	TT	0.042	0.041	0.042	0.040	0.041	0.041	0.042	0.040	0.042
	CT	0.030	0.029	0.030	0.029	0.030	0.030	0.031	0.029	0.031
	BTT	0.042	0.041	0.041	0.040	0.041	0.041	0.042	0.040	0.042

**Table 10:** False positive rate, *n*_*c*_ = *n*_*t*_ = 8, *p* = 5% (3% over and 2% under).

*γ*	Method	*δ*								
		0	0.25	0.50	0.75	1	1.25	1.5	1.75	2
1	PA	0.063	0.060	0.056	0.048	0.042	0.034	0.026	0.019	0.014
	TT	0.047	0.048	0.047	0.047	0.047	0.047	0.050	0.048	0.047
	CT	0.037	0.037	0.036	0.037	0.037	0.037	0.039	0.038	0.037
	BTT	0.047	0.047	0.046	0.047	0.047	0.047	0.050	0.048	0.047

2	PA	0.053	0.051	0.046	0.041	0.032	0.026	0.020	0.015	0.011
	TT	0.047	0.047	0.048	0.050	0.048	0.048	0.048	0.047	0.048
	CT	0.037	0.037	0.036	0.037	0.037	0.037	0.039	0.038	0.037
	BTT	0.047	0.047	0.048	0.049	0.047	0.047	0.048	0.047	0.048

3	PA	0.040	0.039	0.034	0.029	0.023	0.020	0.014	0.011	0.008
	TT	0.047	0.048	0.048	0.049	0.048	0.048	0.048	0.049	0.048
	CT	0.037	0.037	0.038	0.039	0.038	0.038	0.037	0.038	0.038
	BTT	0.047	0.047	0.048	0.049	0.047	0.048	0.048	0.049	0.048

**Table 11:** False positive rate, *n*_*c*_ = *n*_*t*_ = 8, *p* = 10% (7% over and 3% under).

*γ*	Method	*δ*								
		0	0.25	0.50	0.75	1	1.25	1.5	1.75	2
1	PA	0.064	0.059	0.049	0.039	0.027	0.017	0.011	0.006	0.003
	TT	0.049	0.048	0.048	0.049	0.048	0.048	0.049	0.047	0.048
	CT	0.039	0.037	0.038	0.038	0.038	0.038	0.038	0.037	0.037
	BTT	0.049	0.048	0.048	0.049	0.048	0.048	0.048	0.047	0.048

2	PA	0.045	0.041	0.034	0.026	0.019	0.012	0.007	0.004	0.002
	TT	0.048	0.047	0.047	0.047	0.047	0.049	0.048	0.048	0.046
	CT	0.037	0.037	0.036	0.037	0.037	0.039	0.037	0.037	0.037
	BTT	0.048	0.047	0.047	0.047	0.047	0.049	0.047	0.048	0.046

3	PA	0.027	0.024	0.020	0.015	0.010	0.007	0.004	0.002	0.001
	TT	0.048	0.048	0.048	0.046	0.047	0.050	0.047	0.048	0.048
	CT	0.037	0.038	0.037	0.037	0.036	0.039	0.036	0.037	0.038
	BTT	0.048	0.048	0.048	0.047	0.046	0.050	0.047	0.048	0.048

**Table 12:** False positive rate, *n*_*c*_ = *n*_*t*_ = 8, *p* = 20% (5 over and 15 under).

*γ*	Method	*δ*								
		0	0.25	0.50	0.75	1	1.25	1.5	1.75	2
1	PA	0.063	0.058	0.047	0.032	0.021	0.014	0.011	0.006	0.004
	TT	0.048	0.048	0.048	0.048	0.049	0.048	0.048	0.049	0.049
	CT	0.038	0.037	0.037	0.038	0.038	0.038	0.038	0.038	0.038
	BTT	0.048	0.047	0.048	0.048	0.048	0.048	0.048	0.049	0.048

2	PA	0.036	0.036	0.030	0.023	0.017	0.012	0.007	0.004	0.002
	TT	0.047	0.048	0.048	0.048	0.048	0.048	0.048	0.047	0.048
	CT	0.037	0.038	0.037	0.037	0.037	0.037	0.037	0.037	0.038
	BTT	0.047	0.048	0.048	0.048	0.047	0.048	0.047	0.047	0.048

3	PA	0.014	0.017	0.016	0.012	0.010	0.007	0.005	0.002	0.001
	TT	0.048	0.048	0.047	0.048	0.047	0.048	0.047	0.047	0.048
	CT	0.037	0.038	0.036	0.038	0.037	0.038	0.037	0.036	0.038
	BTT	0.048	0.047	0.047	0.048	0.047	0.048	0.047	0.047	0.048

**Table 13:** True discovery rate, *n*_*c*_ = *n*_*t*_ = 4, *p* = 5% (3 over and 2 under).

*γ*	Method	*δ*								
		0	0.25	0.50	0.75	1	1.25	1.5	1.75	2
1	PA	0.053	0.057	0.098	0.155	0.246	0.326	0.420	0.513	0.586
	TT	0.050	0.056	0.083	0.137	0.208	0.269	0.329	0.395	0.444
	CT	0.053	0.060	0.089	0.144	0.224	0.300	0.359	0.439	0.497
	BTT	0.052	0.055	0.084	0.137	0.208	0.272	0.331	0.394	0.442

2	PA	0.185	0.192	0.217	0.267	0.321	0.380	0.463	0.526	0.596
	TT	0.059	0.058	0.074	0.102	0.122	0.148	0.188	0.224	0.262
	CT	0.062	0.068	0.079	0.115	0.145	0.171	0.214	0.268	0.304
	BTT	0.063	0.063	0.075	0.106	0.129	0.154	0.197	0.240	0.277

3	PA	0.325	0.351	0.341	0.394	0.430	0.493	0.533	0.610	0.662
	TT	0.061	0.069	0.071	0.079	0.090	0.118	0.126	0.152	0.182
	CT	0.074	0.082	0.083	0.091	0.111	0.146	0.157	0.191	0.223
	BTT	0.073	0.075	0.083	0.090	0.104	0.131	0.146	0.170	0.202

**Table 14:** True discovery rate, *n*_*c*_ = *n*_*t*_ = 4, *p* = 10% (7 over and 3 under).

*γ*	Method	*δ*								
		0	0.25	0.50	0.75	1	1.25	1.5	1.75	2
1	PA	0.102	0.124	0.180	0.295	0.424	0.564	0.682	0.776	0.857
	TT	0.106	0.131	0.168	0.242	0.338	0.438	0.508	0.577	0.623
	CT	0.107	0.130	0.170	0.261	0.365	0.467	0.547	0.616	0.673
	BTT	0.106	0.132	0.168	0.246	0.343	0.439	0.510	0.579	0.627

2	PA	0.337	0.350	0.406	0.483	0.569	0.661	0.747	0.822	0.877
	TT	0.109	0.127	0.146	0.185	0.224	0.276	0.335	0.389	0.436
	CT	0.115	0.138	0.156	0.207	0.249	0.304	0.370	0.436	0.490
	BTT	0.113	0.132	0.154	0.194	0.231	0.287	0.347	0.408	0.458

3	PA	0.555	0.575	0.619	0.666	0.722	0.785	0.836	0.882	0.922
	TT	0.117	0.134	0.134	0.159	0.179	0.209	0.245	0.281	0.318
	CT	0.135	0.157	0.155	0.181	0.214	0.244	0.295	0.330	0.379
	BTT	0.131	0.147	0.153	0.176	0.200	0.228	0.270	0.309	0.347

**Table 15:** True discovery rate, *n*_*c*_ = *n*_*t*_ = 4, *p* = 20% (5 over and 15 under).

*γ*	Method	*δ*								
		0	0.25	0.50	0.75	1	1.25	1.5	1.75	2
1	PA	0.209	0.235	0.350	0.488	0.629	0.749	0.841	0.903	0.934
	TT	0.209	0.232	0.328	0.432	0.544	0.634	0.699	0.747	0.786
	CT	0.203	0.231	0.336	0.457	0.568	0.661	0.730	0.783	0.817
	BTT	0.207	0.230	0.331	0.434	0.546	0.636	0.704	0.750	0.786

2	PA	0.572	0.575	0.626	0.692	0.743	0.822	0.874	0.919	0.944
	TT	0.229	0.241	0.275	0.340	0.385	0.470	0.536	0.584	0.638
	CT	0.243	0.256	0.289	0.367	0.414	0.514	0.583	0.632	0.684
	BTT	0.235	0.253	0.288	0.350	0.402	0.491	0.552	0.604	0.654

3	PA	0.815	0.817	0.834	0.845	0.870	0.901	0.918	0.947	0.961
	TT	0.237	0.256	0.275	0.303	0.330	0.373	0.423	0.467	0.511
	CT	0.269	0.297	0.318	0.352	0.381	0.431	0.480	0.533	0.577
	BTT	0.257	0.284	0.301	0.331	0.365	0.409	0.452	0.506	0.547

**Table 16:** True discovery rate, *n*_*c*_ = *n*_*t*_ = 8, *p* = 5% (3% over and 2% under).

*γ*	Method	*δ*								
		0	0.25	0.50	0.75	1	1.25	1.5	1.75	2
1	PA	0.048	0.068	0.145	0.262	0.385	0.503	0.619	0.717	0.790
	TT	0.049	0.076	0.141	0.239	0.341	0.414	0.458	0.499	0.517
	CT	0.052	0.075	0.150	0.258	0.371	0.452	0.512	0.549	0.573
	BTT	0.050	0.075	0.141	0.242	0.340	0.415	0.459	0.498	0.519

2	PA	0.179	0.200	0.261	0.331	0.443	0.546	0.638	0.727	0.800
	TT	0.054	0.061	0.087	0.127	0.189	0.249	0.309	0.364	0.407
	CT	0.058	0.067	0.098	0.144	0.212	0.278	0.354	0.412	0.457
	BTT	0.056	0.064	0.093	0.132	0.197	0.259	0.319	0.375	0.416

3	PA	0.324	0.336	0.381	0.450	0.529	0.600	0.699	0.762	0.825
	TT	0.051	0.060	0.073	0.089	0.124	0.162	0.198	0.238	0.286
	CT	0.058	0.069	0.084	0.104	0.148	0.189	0.236	0.278	0.332
	BTT	0.058	0.070	0.078	0.101	0.138	0.176	0.215	0.256	0.304

**Table 17:** True discovery rate, *n*_*c*_ = *n*_*t*_ = 8, *p* = 10% (7% over and 3% under).

*γ*	Method	*δ*								
		0	0.25	0.50	0.75	1	1.25	1.5	1.75	2
1	PA	0.104	0.141	0.276	0.452	0.629	0.777	0.868	0.933	0.970
	TT	0.105	0.140	0.254	0.392	0.507	0.595	0.643	0.678	0.690
	CT	0.106	0.144	0.269	0.420	0.540	0.634	0.688	0.724	0.740
	BTT	0.103	0.140	0.254	0.392	0.508	0.595	0.644	0.680	0.691

2	PA	0.336	0.382	0.467	0.591	0.716	0.821	0.901	0.946	0.973
	TT	0.104	0.122	0.177	0.242	0.334	0.409	0.485	0.542	0.603
	CT	0.112	0.131	0.197	0.268	0.366	0.448	0.532	0.594	0.649
	BTT	0.108	0.129	0.185	0.254	0.344	0.419	0.497	0.552	0.611

3	PA	0.582	0.603	0.657	0.743	0.818	0.882	0.929	0.963	0.984
	TT	0.104	0.112	0.141	0.182	0.232	0.276	0.346	0.398	0.451
	CT	0.121	0.131	0.164	0.210	0.270	0.317	0.395	0.456	0.507
	BTT	0.117	0.126	0.155	0.198	0.253	0.302	0.369	0.423	0.473

**Table 18:** True discovery rate, *n*_*c*_ = *n*_*t*_ = 8, *p* = 20% (5 over and 15 under).

*γ*	Method	*δ*								
		0	0.25	0.50	0.75	1	1.25	1.5	1.75	2
1	PA	0.207	0.283	0.465	0.671	0.820	0.903	0.939	0.969	0.983
	TT	0.213	0.279	0.438	0.591	0.701	0.766	0.805	0.822	0.832
	CT	0.211	0.288	0.456	0.616	0.728	0.793	0.834	0.851	0.863
	BTT	0.212	0.278	0.438	0.592	0.703	0.766	0.806	0.822	0.832

2	PA	0.556	0.591	0.679	0.776	0.856	0.910	0.953	0.975	0.988
	TT	0.211	0.242	0.313	0.420	0.527	0.613	0.679	0.729	0.767
	CT	0.221	0.264	0.341	0.454	0.570	0.653	0.719	0.765	0.801
	BTT	0.223	0.253	0.326	0.432	0.540	0.623	0.691	0.739	0.773

3	PA	0.831	0.817	0.840	0.887	0.915	0.942	0.967	0.984	0.992
	TT	0.213	0.230	0.266	0.334	0.397	0.476	0.547	0.601	0.645
	CT	0.247	0.258	0.302	0.377	0.447	0.527	0.599	0.655	0.693
	BTT	0.237	0.255	0.292	0.361	0.426	0.502	0.574	0.625	0.667
